# TGF-β induces M2-like macrophage polarization via SNAIL-mediated suppression of a pro-inflammatory phenotype

**DOI:** 10.18632/oncotarget.10561

**Published:** 2016-07-13

**Authors:** Fan Zhang, Hongsheng Wang, Xianfeng Wang, Guanmin Jiang, Hao Liu, Ge Zhang, Hao Wang, Rui Fang, Xianzhang Bu, Shaohui Cai, Jun Du

**Affiliations:** ^1^ Department of Pharmacy, Renmin Hospital of Wuhan University, Wuhan 430060, PR China; ^2^ Department of Microbial and Biochemical Pharmacy, School of Pharmaceutical Sciences, Sun Yat-sen University, Guangzhou 510006, PR China; ^3^ Shijiazhuang City Center for Disease Control and Prevention, Shijiazhuang 050000, PR China; ^4^ Department of Clinical Laboratory, Hunan Cancer Hospital and The Affiliated Cancer Hospital of Xiangya School of Medicine, Central South University, Changsha 410013, PR China; ^5^ Cancer Hospital and Cancer Research Institute, Guangzhou Medical University, Guangzhou 510095, PR China; ^6^ Department of Pharmacology, College of Pharmacy, Jinan University, Guangzhou 510632, PR China

**Keywords:** SNAIL, TGF-β, macrophage polarization, tumor-associated macrophage, immunotherapy

## Abstract

Tumor-associated macrophages (TAMs) are a major component of leukocytic infiltrate in tumors, which facilitates tumor progression and promotes inflammation. TGF-β promotes the differentiation of non-activated macrophages into a TAM-like (M2-like) phenotype; however, the underlying mechanisms are not clear. In this study, we found that TGF-β induces a M2-like phenotype characterized by up-regulation of the anti-inflammatory cytokine IL-10, and down-regulation of the pro-inflammatory cytokines TNF-α and IL-12. In human THP-1 macrophages, overexpression of SNAIL caused M2-like differentiation by inhibiting pro-inflammatory cytokine release and promoting the expression of M2-specific markers. By contrast, SNAIL knockdown promoted M1 polarization through up-regulation of pro-inflammatory cytokines and abolished TGF-β-mediated M2-polarization of THP-1 macrophages. The SMAD2/3 and PI3K/AKT signaling pathways were crucial for TGF-β-induced SNAIL overexpression in THP-1 cells. These findings suggest that TGF-β skews macrophage polarization towards a M2-like phenotype via SNAIL up-regulation, and blockade of TGF-β/SNAIL signaling restores the production of pro-inflammatory cytokines. This study provides new understanding of the role of SNAIL in M2 polarization of macrophages, and suggests a potential therapeutic target for antitumor immunity.

## INTRODUCTION

An inflammatory microenvironment is a well-recognized hallmark of cancer progression. Inflammatory circuits can differ considerably between tumors in terms of cellular and cytokine networks and molecular drivers [1]. Macrophages are inflammatory cells that participate in innate immunity and cancer by promoting inflammation [2]. Macrophages display phenotypic and functional plasticity [3], and are divided into two major subsets: classical activation (M1) and alternative activation (M2) [4–6]. Polarized macrophages differ in terms of receptor expression, effector function, and cytokine and chemokine production [4]. Microbial products like lipopolysaccharides (LPS) and interferon-γ drive macrophages towards the M1 phenotype by promoting the Th1 effector response, increasing the expression of pro-inflammatory cytokines and chemokines, and promoting more efficient antigen presentation [5, 7]. In contrast, M2 macrophages (normally stimulated by IL-4/IL-13) display more scavenging activity, express mannose and galactose receptors, have increased phagocytic activity and reduced expression of inflammatory cytokines, and metabolize arginine to ornithine and polyamines via the arginase pathway [8]. These cells inhibit inflammatory response and Th1 immunity, scavenge debris, and promote tumor progression [9, 10].

Tumor associated macrophages (TAMs) are macrophages infiltrating tumor tissues or other tumor-enriched microenvironment [11]. TAMs are mainly M2-like due to exposure to M2 macrophage-differentiation factors produced by the tumor microenvironment [12–14]. Infiltration or enrichment of TAMs is associated with poor prognosis in most human tumor types [15–20]. TAMs participate in circuits that regulate tumor initiation and development, immunosuppression, stroma formation, angiogenesis, invasion, and metastasis [4]. TAMs secrete a plethora of proangiogenic factors, such as vascular endothelial growth factor (VEGF), basic fibroblast growth factor (bFGF), and matrix metalloproteinase-9 (MMP9), which are all associated with tumor angiogenesis and metastasis [4]. TAMs also produce immunosuppressive factors, including interleukin (IL)-10, Indoleamine 2,3-dioxygenase (IDO), and prostaglandin E2, and reduce the production of the proinflammatory cytokine IL-12 [21]. Thus, TAMs influence the efficacy of anticancer chemotherapy [22]. A deeper understanding of the molecular machineries whereby tumor microenvironments regulate the functional plasticity of TAMs may provide useful insights into the development of new therapeutic approaches for reprogramming TAMs toward an M1-like phenotype.

The M2 phenotype is not only induced by IL-4 or IL-13, but also by other stimuli like IL-10, immune complexes, glucocorticoids, and transforming growth factor-β (TGF-β), which leads to variation within the M2-like cells [23]. Known as an immunosuppressive cytokine, TGF-β is overexpressed in tumors and plays an important role in the inhibition of the antitumor immune response and tumor progression [24, 25]. In addition to suppressing lymphocyte function, TGF-β also has an impact on myeloid cell lineages. Recent studies have revealed that TGF-β can suppress or alter the activation, maturation, and differentiation of macrophages, dendritic cells (DCs), and neutrophils [26]. A suppressed innate immune response leads to a weakened adaptive immunity, then immune escape of cancer cells [27]. Stimuli in the tumor environment polarize TAMs towards a pro-tumor M2 rather than an anti-tumor M1 phenotype [28]. TGF-β recruits highly phagocytic TAMs to compete with DCs by suppressing their antigen-presentation, thereby promoting tumor progression [29]. Blockade of TGF-β with inhibitors enhances anti-tumor immunity in patients with cancer. It is conceivable that excessive TGF-β in the tumor microenvironment may block M1 macrophage development, while promoting the alternative activation of M2 macrophages.

The transcription factor SNAIL, expressed in a variety of carcinomas, also plays an important role in tumor progression [30]. Recently SNAIL has been implicated in the regulation of immune suppression and immune tolerance [31]. SNAIL overexpression in skin keratinocytes elicits cytokine and chemokine expression, contributing to the inflammatory microenvironment [32]. In addition, ectopic expression of SNAIL in melanoma cells appears to induce TGF-β and TSP1 expression, by which immunosuppressive nTreg-like cells are generated [33]. These findings suggest a potential regulatory role of SNAIL in cancer-associated inflammation [34]. Here, we show for the first time that the TGF-β/SNAIL signaling pathway is crucial for the differentiation of non-activated macrophages into a TAM-like (M2-like) phenotype, and blockade of this signaling pathway can reverse macrophage polarization from an M2 to an M1-like phenotype.

## RESULTS

### TGF-β induces M2-like macrophage polarization

**Figure 1:**
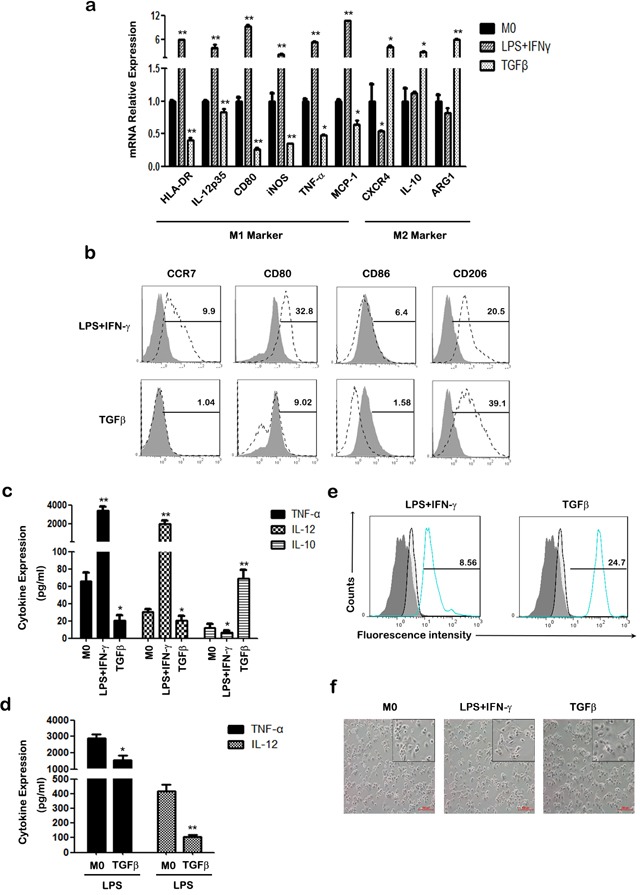
The effect of TGF-β on the polarization of THP-1 macrophages **a.** The mRNA levels of HLA-DR, IL-12p35, CD80, iNOS, TNF-α, MCP-1, CXCR4, IL-10 and ARG1were measured at indicated times by qRT-PCR in M0, M1 and TGF-β induced M2-like macrophages. **b.** The expression of CCR7, CD80, CD86 and CD206 were assessed by FACS using specific mAbs (unfilled histograms) or isotype-matched mAbs (closed histogram). The numbers given for each histogram are the mean fluorescent intensity. **c.** Cell-free supernatants of macrophages were analyzed by ELISA for the production of TNF-α, IL-12 and IL-10. **d.** After treatment with 50 ng/ml LPS for 24 h, the expression of TNF-α and IL-12 in macrophages was analyzed by ELISA. **e.** Phagocytic capacity analysis of macrophages was detected with FITC-latex beads by FACS. Results are shown as histograms representing the mean fluorescent intensity (non-activated macrophages: closed histograms; activated macrophages: unfilled histogram; M1 macrophages (left) or TGF-β induced M2-like macrophages (right): blue line closed histogram) *p < 0.05, **p < 0.01 compared with M0. Results represent mean ± SD of three separate experiments. **f.** Morphology of polarized THP-1 macrophages after treatment with LPS plus IFN-γ or TGF-β for 48h. Images were taken under an inverted microscope (an inset in each panel provides its cropped enlarged image). Scale bars are all equal to 200 μm.

To investigate the effects of TGF-β on macrophage polarization, we evaluated the expression of M1/M2 phenotype markers and inflammatory cytokines in human THP-1 macrophages. In comparison with untreated macrophages (M0), TGF-β treated macrophages exhibited higher mRNA levels of M2 phenotype markers such as CXCR4, IL-10, and arginase 1 (ARG1), while a set of M1 phenotype markers like HLA-DR (MHC II), IL-12p35, CD80, inducible nitric oxide synthase (iNOS), TNF-α and monocyte chemoattractant protein (MCP-1) were down-regulated (Figure 1a). FACS analysis showed that the expression of M1 cell surface markers CCR7 and CD80/CD86 was increased in lipopolysaccharide (LPS) + interferon (IFN)-γ treated macrophages, but decreased in TGF-β treated macrophages (Figure 1b). Accordingly, TGF-β treatment increased CD206 expression of macrophages, which is a classic M2-phenotype marker.

TGF-β stimulated macrophages secreted higher levels of the anti-inflammatory cytokine IL-10, whereas LPS+IFN-γ induced M1 macrophages to produce higher levels of the pro-inflammatory cytokines, TNF-α and IL-12 (Figure 1c). In addition, TGF-β attenuated the expression of the pro-inflammatory cytokines TNF-α and IL-12 in THP-1 macrophages with or without LPS stimulation (Figure 1c&1d). We further investigated phagocytosis of THP-1 macrophages with or without different treatments. Polarized macrophages were incubated with FITC-latex beads, and phagocytic ability examined by FACS. As shown in Figure 1e, TGF-β treated macrophages had an increased percentage of FITC-positive cells, whereas M1 macrophages exhibited lower phagocytic ability. Morphology analysis showed that TGF-β treated macrophages were round cells stretching out pseudopodia, whereas M1 macrophages had elongated fibroblastoid morphology (Figure 1f). These results demonstrate that TGF-β promotes the differentiation of M0 macrophages into M2-like macrophages with diminished pro-inflammatory responsiveness and enhanced anti-inflammatory ability.

### SNAIL overexpression promotes M2 polarization of macrophages

**Figure 2:**
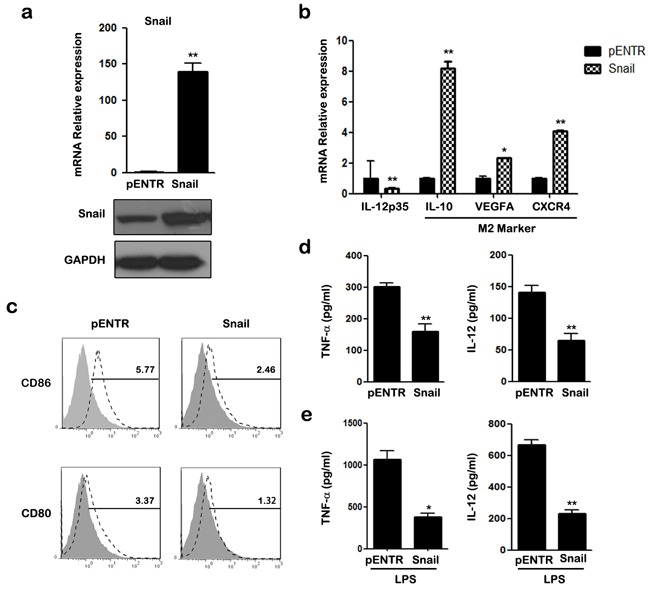
Overexpression of SNAIL in THP-1 macrophages mediates the inhibitory effect of M1 polarization **a.** Cells were analyzed for mRNA and protein expression of SNAIL by qRT-PCR and western blot. **b.** qRT-PCR analysis of mRNA for IL-12p35, IL-10, VEGFA and CXCR4 in transfected macrophages. **c.** The expression of CD86 and CD80 was assessed by FACS using specific mAbs (unfilled histograms) or isotype-matched mAbs (closed histogram). The numbers given for each histogram are the mean fluorescent intensity. **d.** The secretion of TNF-α and IL-12 in transfected macrophages was detected by ELISA. **e.** ELISA analysis shown cytokine TNF-α and IL-12 production after 24 h treatment with LPS. * *p* < 0.05, ** *p* < 0.01 compared with control. Results represent mean ± SD of three separate experiments.

SNAIL which is a well-known TGF-β-sensitive transcription factor, is implicated in the regulation of immune suppression and immune tolerance [31]. Therefore, we investigated whether SNAIL was involved in TGF-β induced M2-like polarization of THP-1 macrophages. Transcription and expression of SNAIL were detected with qRT-PCR and western blotting (Figure 2a). As shown in Figure 2b, transfection of pcDNA-SNAIL increased expression of M2-associated genes IL-10, VEGFA, and CXCR4, but decreased expression of the M1-associated gene IL-12p35. Furthermore, FACS analysis showed that the expression of CD80/CD86 was lower in macrophages after SNAIL overexpression (Figure 2c). Alternatively activated macrophages are characterized with lower expression of pro-inflammatory mediators and attenuated response to pro-inflammatory stimuli. Thus, we measured the release of the pro-inflammatory cytokines, TNF-α and IL-12, in supernatants of THP-1 macrophages. THP-1 cells with SNAIL overexpression had lower TNF-α and IL-12 production with or without LPS stimulation (Figure 2d&2e). These data reveal that overexpression of SNAIL in THP-1 cells induces an M2-like phenotype polarization, while suppressing an M1-like phenotype.

### Silencing of SNAIL results in M1 polarization of macrophages

**Figure 3:**
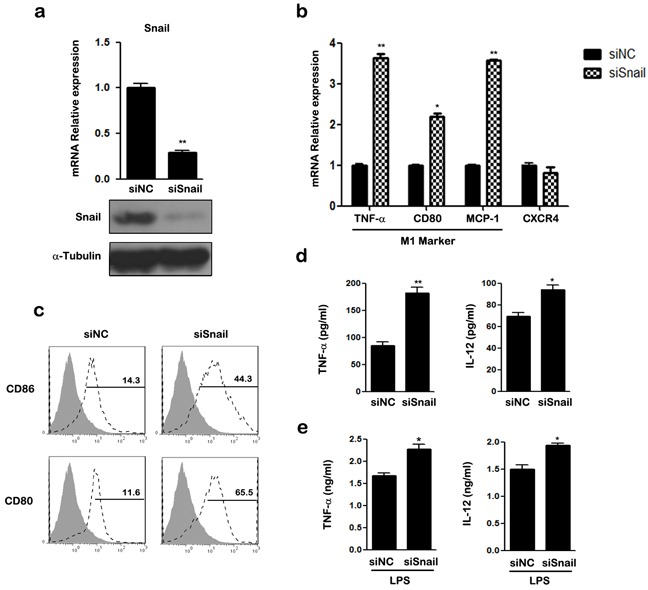
Knockdown of SNAIL in THP-1 macrophages promotes M1 and suppresses M2 polarization **a.** Expression of SNAIL was examined by qRT-PCR and western blot. **b.** The mRNAs of TNF-α, CD80, MCP-1 and CXCR4 were detected by qRT-PCR in siRNA transfected THP1 macrophages. **c.** The expression of CD86 and CD80 was assessed by FACS using specific mAbs (unfilled histograms) or isotype-matched mAbs (closed histogram). The numbers given for each histogram are the mean fluorescent intensity. **d.** Cell-free culture supernatants were determined by ELISA for the generation of TNF-α and IL-12. **e.** ELISA analysis of TNF-α and IL-12 by siRNA transfected macrophages and then left stimulated with LPS for 24 h. * *p* < 0.05 and ** *p* < 0.01 compared with control. Results represent mean ± SD of three separate experiments.

We next silenced SNAIL expression using small interfering RNA (siRNA). The mRNA and protein expression of SNAIL were examined using qRT-PCR and western blotting (Figure 3a). As shown in Figure 3b, silencing of SNAIL up-regulated the expression of M1-associated genes TNF-α, CD80, and MCP-1, and attenuated the expression of CXCR4 in THP-1 macrophages. Knockdown of SNAIL enhanced the expression of the M1 cell surface markers CD80, CD86, and HLA-DR (data not shown; Figure 3c). Compared to the control group, production of TNF-α and IL-12 was increased in SNAIL-knockdown (siSNAIL) macrophages (Figure 3d). After stimulation with LPS for 24 h, silencing of SNAIL also promoted the expression of pro-inflammatory cytokines (Figure 3e). Collectively, these observations suggest that knockdown of SNAIL skews macrophages toward a pro-inflammatory M1 phenotype.

### SNAIL is critically involved in TGF-β induced M2-like polarization

**Figure 4:**
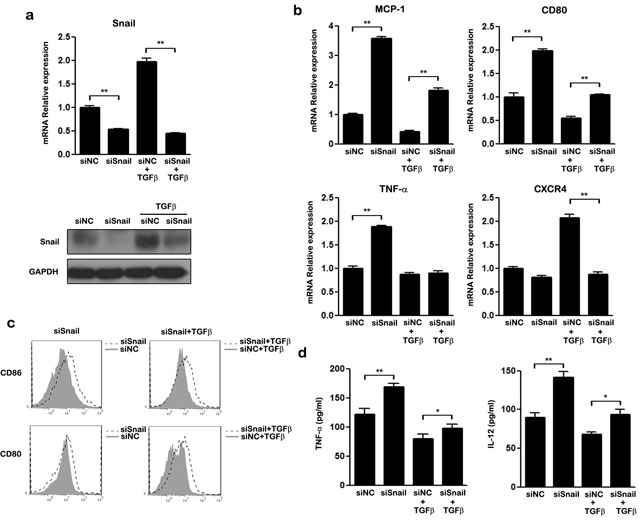
The effect of SNAIL on TGF-β mediated M2-like polarization in THP-1 macrophages THP-1 macrophages transfected with siNC or siSNAIL were stimulated with or without TGF-β for 48 h. **a.** Expression of SNAIL was examined by qRT-PCR and western blot. **b.** The mRNA levels of MCP-1, CD80, TNF-α and CXCR4 in treated macrophages were detected by qRT-PCR. **c.** FACS analysis of CD86 and CD80 expression on siRNA transfected macrophages after treatment with (right panel) or without TGF-β (left panel). Macrophages transfected with SNAIL siRNA: unfilled histogram; macrophages transfected with negative control siRNA: closed histograms. **d.** ELISA analysis of the secretion of TNF-α and IL-12 by macrophages transfected with siRNA either in the presence of TGF-β or not. * *p* < 0.05 and ** *p* < 0.01 compared with control. Results represent mean ± SD of three separate experiments.

TGF-β is a direct enhancer of SNAIL expression [35]. We next investigated whether SNAIL is involved in the TGFβ induced M2-like polarization of THP-1 macrophages. Our results confirmed that, compared to untreated cells, TGF-β increased both the mRNA and protein levels of SNAIL in THP-1 cells (Figure 4a). We performed gene knockdown assays to verify whether SNAIL is a key regulator in TGF-β mediated M2-like polarization. As shown in Figure 4a, when SNAIL was silenced by siRNA, even TGF-β treatment could not increase the expression of SNAIL. TGF-β treatment also promoted M2 polarization by down-regulating MCP-1, CD80, and TNF-α mRNA and inhibiting expression of the cell surface markers CD86 and CD80, consistent with previous data (Figure 4b&4c). Silencing of SNAIL also attenuated TGF-β induced down-regulation of TNF-α and IL-12 expression when compared with the control group (Figure 4d). These findings demonstrate that SNAIL is essential for TGF-β induced M2-like polarization in THP-1 macrophages.

### SNAIL is also critical for TGF-β induced M2-like polarization of BMDMs

**Figure 5:**
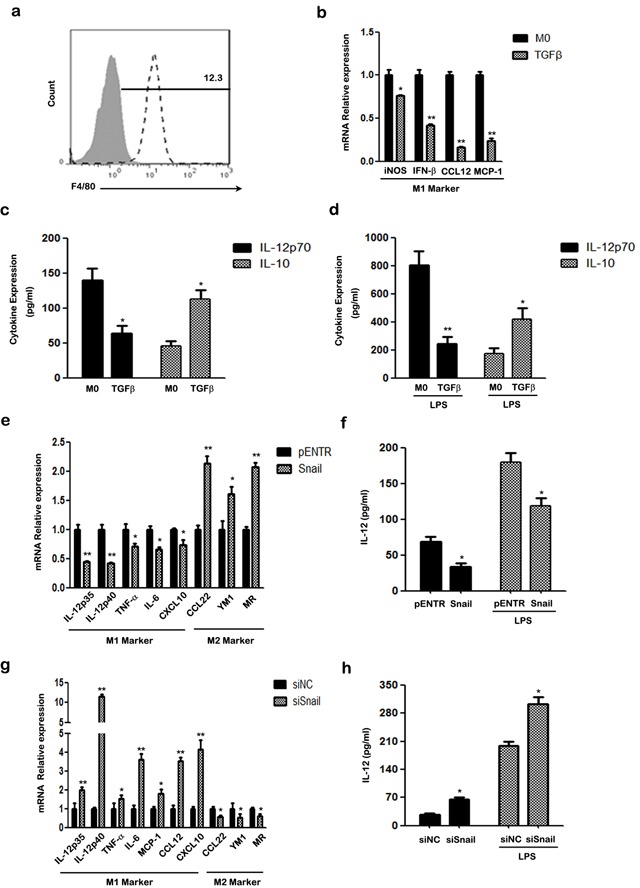
The effect of TGF-β/SNAIL on the polarization of BMDMs **a.** FACS analysis of cell surface marker F4/80 in BMDMs using specific mAbs (unfilled histograms) or isotype-matched mAbs (closed histogram). **b.** The mRNA levels of iNOS, INF-β, CCL12, MCP-1 of treated BMDMs were detected by qRT-PCR. **c.** Secretion of murine IL-12p70 and IL-10 was examined using ELISA. **d.** ELISA analysis of murine IL-12p70 and IL-10 production in treated BMDMs, after treatment with LPS for another 24 h. **e.** The mRNA levels of M1/2 hallmarks IL-12p35, IL-12p40, TNF-α, IL-6, CXCL10, CCL22, YM1, MR in SNAIL overexpression BMDMs were detected by qRT-PCR. **f.** ELISA analysis of murine IL-12p70 production in supernatant of SNAIL transfected BMDMs with or without LPS stimulation for another 24 h. **g.** The mRNA levels of the M1-specific markers IL-12p35, IL-12p40, TNF-α, IL-6, MCP-1, CCL12, CXCL10 and M2-specific markers CCL22, YM1 and MR in SNAIL-silencing BMDMs were determined by qRT-PCR. **h.** The production of murine IL-12p70 of SNAIL-silencing BMDMs with or without LPS stimulation was measured by ELISA. * *p* < 0.05 and ** *p* < 0.01 compared with control. Results represent mean ± SD of three separate experiments.

We next used bone marrow-derived macrophages (BMDMs) to verify that the TGF-β/SNAIL pathway is crucial for M2-like polarization in primary cells. BMDMs were pre-matured with murine M-CSF for 5 days and the common mouse macrophage marker F4/80 was examined by FACS (Figure 5a). Then, BMDMs were stimulated with TGF-β, and the M1/M2 phenotype was detected. As expected, TGF-β inhibited M1 polarization and promoted M2 polarization of BMDMs. TGF-β inhibited M1-specific expression of iNOS, IFN-β, CCL12 and MCP-1 in differentiated BMDMs (Figure 5b). The BMDMs also secreted less IL-12p70 and more IL-10 in response to TGF-β stimulation, whether or not the cells were stimulated with LPS (Figure 5c&5d).

Next, we ectopically expressed SNAIL to verify the effects of SNAIL on macrophage polarization. M2 markers, such as CCL22, YM1, and MR were more highly expressed, whereas M1-specific genes IL-12p35, IL-12p40, TNF-α, IL-6, CXCL10 were down-regulated in macrophages with SNAIL overexpression (Figure 5e). In addition, SNAIL-transfected BMDMs secreted less IL-12p70 even after stimulation with LPS (Figure 5f).

Finally, we silenced SNAIL in BMDMs using specific targeting SNAIL siRNA. As shown in Figure 5g, knockdown of SNAIL caused increased expression of IL-12p35, IL-12p40, TNF-α, IL-6, MCP-1, CCL12 and CXCL10, which are classic markers of the M1 phenotype. In contrast, higher expression of the pro-inflammatory cytokine IL-12p70 was observed in SNAIL silenced macrophages than in the control group (Figure 5h). Taken together, our results indicate that SNAIL is important for M2-polarization of BMDMs.

### TGF-β up-regulates SNAIL protein via Smad2/3 and AKT signaling pathways

**Figure 6:**
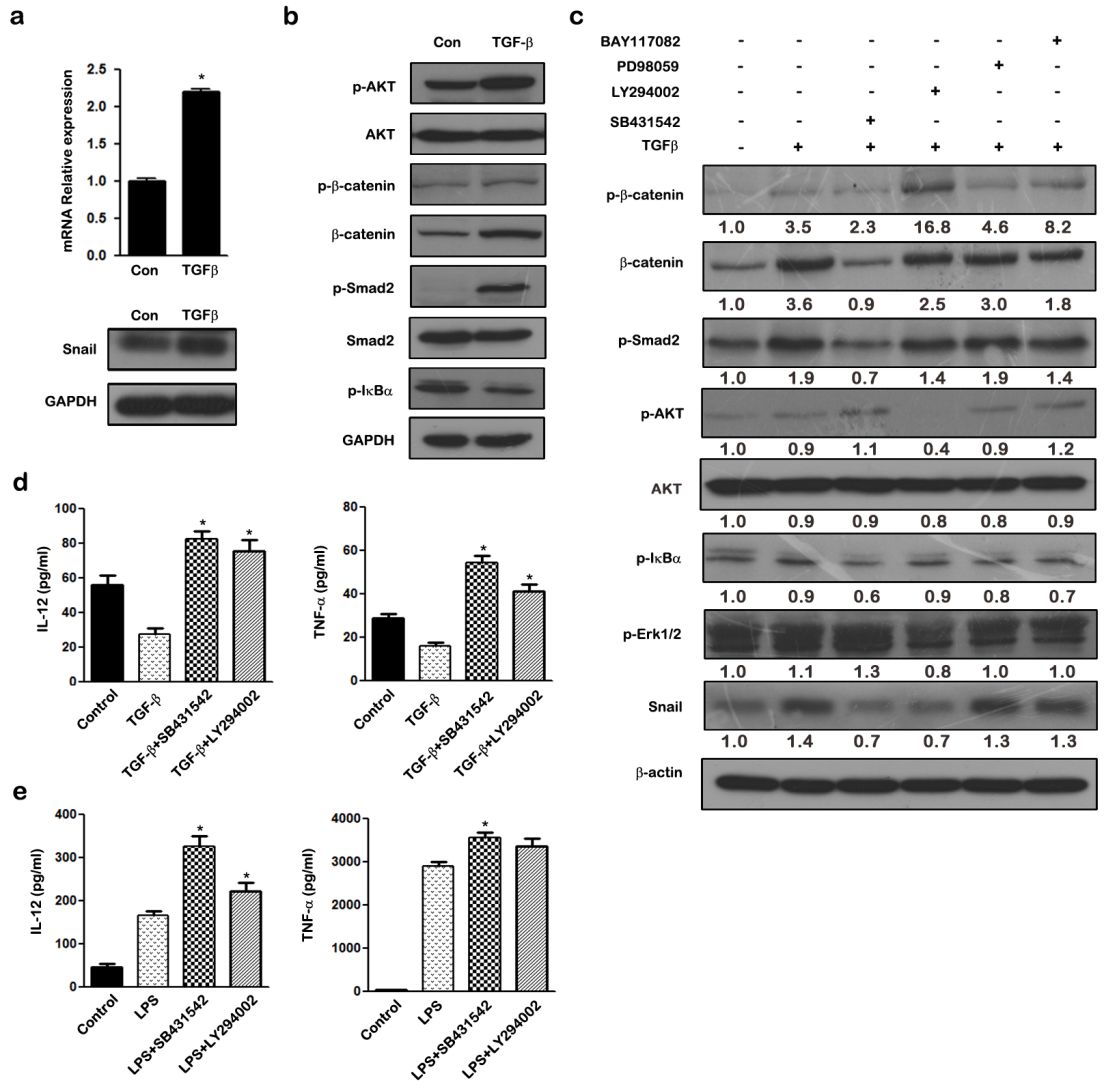
TGF-β regulates SNAIL expression via activation of PI3K/AKT and Smad2/3 signaling pathways in THP-1 macrophages **a.** qRT-PCR and western blot analysis of SNAIL in TGF-β treated THP-1 macrophages. **b.** THP-1 macrophages were stimulated for 24 h with TGF-β, and the cell lysate were subjected to western blotting with anti-p-AKT, AKT, p-β-catenin, β-catenin, p-Smad2, Smad2, p-IκBα and GAPDH antibodies. **c.** THP-1 macrophages were pretreated with LY294002 (10 μM), SB431542 (20 μM), PD98059 (10 μM), BAY11-7082 (10 μM) for 1 h respectively followed by stimulation with TGF-β for 24 h. The expression of SNAIL, p-β-catenin, p-AKT, AKT, p-Smad2, p-IκBα, and p-Erk1/2 were examined by western blot. **d.** Cytokine production by macrophages pretreated for 1 h with medium, 20 μM SB431542, 10 μM LY294002 and left unstimulated or stimulated with TGF-β for 24 h. Cell-free supernatants were analyzed by ELISA for production of TNF-α and IL-12. **e.** Cytokine production in macrophages pre-incubated for 1 h with medium only, 20 μM SB431542, 10 μM LY294002 and left unstimulated or stimulated with LPS. Cell-free supernatants were analyzed by ELISA for production of TNF-α and IL-12. * *p* < 0.05 and ** *p* < 0.01 compared with control. Results represent mean ± SD of three separate experiments.

As shown in Figure 6a, TGF-β stimulation induced the mRNA and protein expression of SNAIL in THP-1 macrophages. The PI3K/AKT, Smad2/3, NF-κB, and MAPK pathways were all activated upon TGF-β stimulation in THP-1 macrophages (Figure 6b). Inhibitors of NF-κB (BAY11-7082), PI3K/AKT (LY294002), ERK (PD98059), and ALK5 (SB431542) were used to investigate the molecular mechanisms underlying TGF-β mediated SNAIL expression. THP-1 macrophages were pretreated with inhibitors for 1 h before TGF-β stimulation, and then the expression of SNAIL and pathway molecules were determined by western blotting. Both the PI3K/AKT inhibitor (LY294002) and ALK5 inhibitor (SB431542) completely blocked TGF-β induced SNAIL up-regulation, whereas the NF-κB and ERK pathways did not influence SNAIL expression (Figure 6c). Macrophages treated with LY294002 showed phosphorylation of β-catenin, thus demonstrating its ability to enhance GSK3β activity. These results suggest that TGF-β mediated SNAIL expression occurs mainly via the PI3K/AKT and Smad2/3 pathways.

In addition, we assessed the effect of blocking the TGF-β/SNAIL pathway on macrophage polarization. Inhibition of TGF-β/SNAIL using either LY294002 or SB431542 blocked the ability of TGF-β to skew M2-like polarization, and resulted in decreased expression of pro-inflammatory cytokines TNF-α and IL-12 (Figure 6d). In contrast, expression of TNF-α and IL-12 was increased when THP-1 macrophages were stimulated with LPS in the presence of LY294002 or SB431542 compared with the LPS-stimulated group (Figure 6e). These data demonstrate that blockade of either the PI3K/AKT or the Smad2/3 pathway reverses the immunophenotype of TGF-β induced macrophages from an anti-inflammatory M2-like phenotype to a pro-inflammatory M1 phenotype.

## DISCUSSION

Plasticity and diversity are key features of the monocyte-macrophage differentiation pathway [6]. Orchestration of macrophage cell function is an important element in pathways connecting inflammation and cancer [36]. Different macrophage subtypes perform specific functions [37]. M1 and M2 macrophages have distinct cytokine profiles, with M1 macrophages expressing high levels of pro-inflammatory factors and co-stimulatory molecules, while M2 macrophages are characterized by higher expression of M2 markers and enhanced phagocytic capacity but lower expression of HLA-DR and pro-inflammatory cytokines [10, 38–40]. The mechanisms of M1-M2 switches, which may involve the recruitment of circulating precursors or the reeducation of cells, remains unclear *in vivo*.

TGF-β is a pleiotropic cytokine that exerts dual roles in cancer, from tumor suppressor in less advanced tumors and healthy cells to a pro-metastatic molecule in more aggressive cancers [41]. In tumor development, immunosuppressive cells such as tumor associated macrophages (TAMs) and myeloid-derived suppressor cells (MDSCs) can produce high levels of TGF-β to support tumor growth, metastasis, and immune escape [42]. TGF-β is probably a major proximal cytokine within tumors and likely affects macrophage function. In our study, TGF-β increased expression of the anti-inflammatory cytokine IL-10 and mannose receptor CD206 in macrophages and mediated their strong pro-phagocytic activity. Moreover, expression of the pro-inflammatory factor TNF-α, IL-12, and the co-stimulatory molecules CD80, CD86, and CCR7 were decreased in THP-1 macrophages in response to TGF-β. Likewise, TGF-β also stimulated murine BMDMs to display an M2-like phenotype characterized by high levels of IL-10 and low levels of IL-12p70, and M1-specific markers. Our findings thus demonstrate that TGF-β promotes macrophage polarization toward a M2-like phenotype.

The tumor-derived factor, TGF-β, favors the differentiation of M2 versus M1 macrophages and may regulate NF-κB and C/EBP expression and activation in tumor-bearing hosts [43, 44]. Nonetheless, other immunosuppressive mechanisms may be also involved. Our study indicates that TGF-β mediates M2 polarization through up-regulation of the transcription factor, SNAIL. Knockdown of SNAIL by siRNA abolishes TGF-β induced phenotypic changes and partly restores the pro-inflammatory cytokine expression in THP-1 macrophages, suggesting that SNAIL expression is important for TGF-β-mediated M2 polarization.

SNAIL is a pleiotropic transcription factor which acts as a key inducer of epithelial-mesenchymal transition (EMT) and also plays an important role in a variety of biologic processes, including cell survival, immune regulation, and stem cell biology [31]. SNAIL simultaneously induces cancer EMT and immunosuppression in a variety of human cancers [31, 33, 45]. The data presented here show that SNAIL is up-regulated in TGF-β alternative activated macrophages with typical M2 features. Overexpression of SNAIL in either human THP-1 macrophages or murine BMDMs suppresses the inflammatory M1 phenotype and skews macrophages toward a M2 phenotype. SNAIL-overexpressed macrophages polarize toward the M2 phenotype by reducing expression of IL-12p70, TNF-α, and a series of M1-specific genes. Inhibition of SNAIL results in a substantial increase in production of the pro-inflammatory mediators, TNF-α and IL-12, after TLR stimulation, whereas the expression of IL-10 is not altered (data not shown). In addition to the direct effect of these cytokines, macrophages overexpressing SNAIL have lower expression of the co-stimulatory molecules, CD80 and CD86, which are important for the induction of T cells. We also demonstrated that SNAIL regulates the expression of genes with a relevant role in M2 alternative activation. These results reveal an important role for SNAIL in regulating M2-like polarization, and will hopefully provide us with novel therapeutic approaches for treating cancer by reprogramming TAMs towards M1 tumoricidal cells.

In addition to these findings, we also showed that SNAIL is a rapid responder to TGF-β stimulation. Previous studies have demonstrated that expression of SNAIL is regulated by a complex signaling network at the transcriptional and post-transcriptional levels in various tumor cells [46, 47]. This network includes not only the canonical Smad2/3 pathway, but also the phosphatidylinositol 3-kinase (PI3K), the mitogen-activated protein kinase (MAPKs), the glycogen synthase kinase 3-beta (GSK-3β), and the NF-κB pathways [48, 49]. The TGF-β/Smad pathway induces SNAIL expression by directly binding to SNAIL promoter, while PI3K/AKT signaling regulates the expression of SNAIL protein by modulating the activity of GSK-3β [50]. Phosphorylation of GSK-3β maintains SNAIL in an inactive state thereby increasing its stability. We found that TGF-β induced SNAIL expression was blocked by treatment with the PI3K inhibitor LY294002 and the ALK/Smad inhibitor, SB431542, suggesting that the PI3K/AKT and Smad2/3 signaling pathways are essential for TGF-β-induced SNAIL expression in THP-1 macrophages and that SNAIL expression is regulated by complex molecular mechanisms.

In summary, we are the first to show that TGF-β modulates the polarization of macrophages to an M2-like phenotype, which is important in the promotion of tumor progression and invasion. We further provide evidence that SNAIL is a key regulator involved in M2 alternative activation induced by TGF-β, and SNAIL expression is controlled by the PI3K/AKT and Smad2/3 signaling pathways. Our findings identify a critical role of SNAIL in modulating anti- versus pro-inflammatory cytokines and demonstrate that SNAIL promotes M2 polarization in both human and murine macrophages. This study provides a novel understanding of SNAIL in M2 polarization of macrophages, and suggests a potential therapeutic target for more effective antitumor immunity.

## MATERIALS AND METHODS

### Reagents

Medium, supplements, and fetal bovine serum (FBS) for cell culture were purchased from GIBCO BRL (Grand Island, N.Y.). Small molecule inhibiting agents: ALK5 inhibitor SB431542, PI3K inhibitor LY294002, NF-κB inhibitor BAY11-7082, ERK inhibitor PD98059 were obtained from Beyotime. Lipopolysaccharide (LPS), phorbol-12-myristate-13 acetate (PMA) and FITC-latex beads were purchased from Sigma-Aldrich (St Louis, MO). Primary antibodies against SNAIL, p-Akt (Ser473), Akt, p-Smad2, Smad2, p-Erk1/2, p-β-catenin, p-IκBα were purchased from Cell Signaling Technology (MA, USA) and anti-β-catenin, β-actin, α-Tubulin, GAPDH were from Santa Cruz Biotechnology (Santa Cruz, CA, USA). FITC- or PE-conjugated antibodies for flow cytometry were supplied as follows: F4/80, CD80, CD86, from eBioscience (San Diego, CA); CCR7, CD206 from BD Biosciences (San Jose, CA). Lipofectamine 2000 was purchased from Invitrogen (Carlsbad, CA, USA). Recombinant protein human and mouse TGF-β1, human IFN-γ, and murine macrophage colony stimulating factor (M-CSF) were bought from Pepro Tech (Rocky Hill, NJ). PrimeScript^®^ RT reagent Kit and SYBR^®^ Premix Ex Taq™ were products of TaKaRa. E.Z.N.Z^®^ HP Total RNA Kit was bought from Omega Bio-Tek (Norcross, CA, USA).

### Cell culture

THP-1 monocytes were cultured with RPMI1640 supplemented with 10% FBS and 2 mmol/l L-glutamine under a humidified 5% CO_2_ atmosphere at 37°C. For active participation of macrophages in the inflammatory and immune responses, the monocytes were exposed to PMA (20 ng/ml) for 24 h and the medium was replaced 1 h before the next treatment. Cells were treated for 48 h with normal medium to be M0, with a polarizing mix of LPS (100 ng/ml) and IFN-γ (50 ng/ml) to be M1, and with TGF-β1 (20 ng/ml) to become M2-like.

Balb/C mice aged 6–8 weeks were purchased from the Medical Experimental Animal Center of Guangdong Province (China). Bone marrow was flushed from femurs and tibias of Balb/C mice. Bone marrow-derived monocytes (BMDMs) were collected with Hanks' balanced salt solution (HBSS). The cell suspension was then centrifuged and the pellet was resuspended in RPMI1640 medium containing 10% FBS, 2 mmol/l L-glutamine, and 20 ng/ml murine macrophage colony stimulating factor (M-CSF) for differentiation to macrophages. Cells were then cultured for 5 days and non-adherent cells were removed. More than 80% of the adherent cells acquired were F4/80^+^ macrophages (as determined by fluorescence-activated cell sorter (FACS) analysis) and these cells were used for M0 macrophages. A total of 1×10^6^ cells/ml BMDMs were dispensed into 6-well cell culture plates (Nunc, USA) and stimulated with 20 ng/ml recombinant mouse TGF-β1 with or without 50 ng/ml LPS.

### Cell transfection

The human SNAIL expression plasmid pcDNA-SNAIL, pcDNA3.1 control vector and mouse cDNA plasmid pCMV-SNAIL or pCMV6 empty vector (OriGene Technologies), were used for SNAIL over-expression. Scrambled siRNA or specific siRNA targeting human/murine SNAIL (RiboBio Co Ltd, China) were used for SNAIL knockdown in THP-1 macrophages or BMDMs. Before transfection, the cells were seeded on a 6-well plate (2×10^6^/well) and cultured for 24h. Cells were then transfected with 2 μg plasmid vector or 100 pmol siRNA oligomer mixed with lipofectamine 2000 reagent in serum-free OPTI-MEM (Invitrogen, Germany) according to the manufacturer's instructions. Medium was changed to complete culture medium 6 h later, and the cells were incubated at 37°C in a CO_2_ incubator for another 24 to 48 h before harvest.

### Quantitative real-time PCR

Total RNA was extracted using E.Z.N.Z^®^ HP Total RNA Kit (Omega Bio-tek, USA) after treatments. Quantitative real-time PCR (qRT-PCR) assays were performed in a LightCycler 480 instrument (Roche Diagnostics, Switzerland) using SYBR^®^ Premix Ex Taq™ (Takara Bio). Results were normalized according to the expression of *GAPDH* mRNA. After normalization, expression of each target gene was calculated using the comparative threshold cycle (CT) method. Data are presented as the mean ± standard deviation (SD) from three independent experiments. The PCR primer sequences obtained from Sangon Biotech (Shanghai, China) are presented in Supplementary Table S1 and S2.

### Measurement of cytokine production

The secretion of human TNF-α, IL-12, and IL-10 were quantified by a cytometric bead array (Th1/Th2 Cytokine CBA, BD Pharmingen) according to manufacturer's instructions. Mouse IL-12p70 and mouse IL-10 levels in the supernatants of cultured macrophages were measured by sandwich enzyme-linked immunosorbent assay (ELISA) using a commercially available ELISA kit (eBioscience), as described by the manufacturer.

### Western blot analysis

Whole cell lysates were harvested and western blot analysis was performed as described previously [30]. Primary antibodies included anti-SNAIL, anti-p-AKT, anti-AKT, anti- p-β-catenin, anti-β-catenin, anti-p-Smad2, anti-Smad2, anti-p-IκBα, anti-p-Erk1/2, anti-α-Tubulin, anti-GAPDH and anti-β-actin. Secondary antibodies included goat anti-rabbit/mouse IgG-HRP (Bioworld, China). All western blots were visualized with ECL western blotting substrate (Pierce, USA). Results of densitometric analyses of western blots, obtained using Image-Pro plus software, are presented as relative optical density to the control (β-actin).

### Flow cytometry analysis

For analysis of macrophage surface antigen expression, PE- and FITC-conjugated Abs anti-human CCR7, CD80, CD86, CD206 and anti-mouse F4/80 were used. Mouse and rat isotype controls were also used. The cells were washed and stained for 30 min at 4°C with the optimal dilution of each antibody. Cells were washed again and analyzed by flow cytometry (EXL™, Beckman Coulter). Data analysis was done using FlowJo software (Ashland, OR). All assays were repeated at least three independent times.

### Statistical analysis

Results are expressed as Mean ± SD of three independent experiments unless otherwise specified. Data were analyzed by two-tailed unpaired Student's *t*-test between any two groups and by One-Way ANOVA followed by Bonferroni tests for multiple comparisons. Statistical analysis was carried out using GraphPad Prism Software Version 5.0 (GraphPad Software Inc., La Jolla, CA). Flowjo software A (Ashland, OR) was used to analyze the data of flow cytometry. A *p*-value of <0.05 was considered statistically significant.

## SUPPLEMENTARY TABLES


