# Supplementary material for: TGF-β induces M2-like macrophage polarization via SNAIL-mediated suppression of a pro-inflammatory phenotype
Source: Oncotarget. 2016 Jul 13;7(32):52294–306. doi: 10.18632/oncotarget.10561 (PMC5239552; doi:10.18632/oncotarget.10561)
Supplement: Supplementary file 1 [file oncotarget-07-52294-s001.pdf]

TGF-β induces M2-like macrophage polarization via SNAIL-mediated suppression of a pro-inflammatory phenotype

SUPPLEMENTARY TABLES

Supplementary Table S1: Real-Time primers of human genes

Gene	Forward primer	Reverse primer
HLA-DR	5′- GCCCAACCTGGAAATCATGACA-3′	5′- AGGGCTGTTGTGAGCACA-3′
IL-12p35	5′- GATGAGCTGATGCAGGCC-3′	5′- AGTCCTCCACCTCGTTGTCCGTGA-3′
CD80	5′- GGGAAAGTGTACGCCCTGTA-3′	5′- GCTACTTCTGTGCCCACCAT-3′
iNOS	5′- AGCTGAACTTGAGCGAGGAG-3′	5′- GGAAAAGACTGCACCGAAGA-3′
TNF-α	5′- CCCCAGGGCTCCAGAAGGT-3′	5′- TGGGGCAGAGGGTTGATTAGTTG-3′
MCP-1	5′- AAGATCTCAGTGCAGAGGCTCG-3′	5′- CACAGATCTCCTTGGCCACAA-3′
CXCR4	5′- CACTTCAGATAACTACACCG-3′	5′- ATCCAGACGCCAACATAGAC-3′
IL-10	5′- AACAAGAGCAAGGCCGTGG-3′	5′- GAAGATGTCAAACTCACTCATGGC-3′
ARG1	5′- -CAGATATGCAGGGAGTCACC-3′	5′- CAGAAGAATGGAAGAGTCAG-3′
VEGFA	5′- GAAGAAGCAGCCCATGACAG-3′	5′- GATCCTGCCCTGTCTCTCTG-3′
Snail	5′- GACCACTATGCCGCGCTCTT-3′	5′- TCGCTGTAGTTAGGCTTCCGATT-3′
GAPDH	5′- GCACCGTCAAGGCTGAGAAC-3′	5′- TGGTGAAGACGCCAGTGGA-3′

Notes: IL, interleukin; iNOS, inducible nitric oxide synthase; TNF-α, tumor necrosis factor alpha; MCP-1, monocyte chemoattractant protein 1; CXCR4, chemokine CXC chemokine receptor 4; VEGFA, vascular endothelial growth factor A; ARG1, arginase 1; GAPDH, glyceraldehyde-3-phosphate dehydrogenase.

Supplementary Table S2: Real-Time primers of murine genes

Gene	Forward primer	Reverse primer
iNOS	5′- GTTCTCAGCCCAACAATACAAGA-3′	5′- GTGGACGGGTCGATGTCAC-3′
IFN-β	5′- CCCTATGGAGATGACGGAGA-3′	5′- CTGTCTGCTGGTGGAGTTCA-3′
TNF-α	5′- CGGTGCCTATGTCTCAGCCT-3′	5′- GAGGGTCTGGGCCATAGAAC-3′
IL-6	5′- AGTTGCCTTCTTGGGACTGA-3′	5′- TCCACGATTTCCCAGAGAAC-3′
IL-12p35	5′- CTGTGCCTTGGTAGCATCTA-3′	5′- TTTCACTCTGTAAGGGTCTG-3′
IL-12p40	5′- AGGTGCGTTCCTCGTAGAGA-3′	5′- AAAGCCAACCAAGCAGAAGA-3′
MCP-1	5′- ATCCCAATGAGTAGGCTGGAGAGC-3′	5′- CAGAAGTGCTTGAGGTGGTTGTG-3′
CXCL10	5′- CGGAATCTAAGACCATCAA-3′	5′- TCACCTTTCAGAAGACCAA-3′
CCL12	5′- ACTTCTATGCCTCCTGCTC-3′	5′- CACTGGCTGCTTGTGATTC-3′
CCL22	5′- ATTACGTCCGTTACCGTCTG-3′	5′- TAGGCTCTTCATTGGCTCAG-3′
YM1	5′- TTATCCTGAGTGACCCTTCTAAG-3′	5′- TCATTACCCTGATAGGCATAGG-3′
MR	5′- GCTGAATCCCAGAAATTCCGC-3′	5′- ATCACAGGCATACAGGGTGAC-3′
Snail	5′- TCTGAAGATGCACATCCGAGC-3′	5′- TTGCAGTGGGAGCAGGAGAAT-3′
GAPDH	5′- TGTGTCCGTCGTGGATCTGA-3′	5′- TTGCTGTTGAAGTCGCAGGAG-3′

Notes: iNOS, inducible nitric oxide synthase; IFN, interferon; TNF-α, tumor necrosis factor alpha; IL, interleukin; MCP-1, monocyte chemoattractant protein 1; CXCL, chemokine CXC chemokine ligand; CCL, chemokine (C-C motif) ligand; MR, mannose receptor; GAPDH, glyceraldehyde-3-phosphate dehydrogenase.
